# Dr. Edward Watrous Jenks

**Published:** 1903-05

**Authors:** 


Dr. Edward Watrous Jenks, of Detroit, died March 19, 1903, aboard a railway train on which he was returning from Mexico. He contracted pneumonia en route and died suddenly. Dr. Jenks was one of the distinguished physicians of the country and a most genial personality, who will be widely missed and mourned.

